# On One of the Functions Performed by the Liver, More Particularly in the Fœtus, and in Amphibious Animals

**Published:** 1828-09

**Authors:** Egerton A. Jennings

**Affiliations:** Member of the Royal College of Surgeons.


### PHYSIOLOGY.

*On one of the Functions performed by the Liver, more particularly in the Fœtus, and in Amphibious Animals.* By Egerton A. Jennings, Member of the Royal College of Surgeons.

In this paper I shall endeavour to shew that the liver, like the lungs, possesses the power of *decarbonising the blood.* I am aware that other persons have entertained a similar opinion, but several facts are here detailed which have not been, I believe, previously observed; or, at least, have not been publicly stated.*

If we examine the circulating system in different animals, we shall find some that require the blood to be constantly exposed to the influence of oxygen; others that can support life for a considerable time without such exposure; and a third class that live without ever having their circulating fluid exposed to its influence.

In the first class, or those that require a constant supply of oxygen, the greatest diversity is observable in the apparatus

* See Ornithologia, by J. Jennings, page 55.

by which the blood is exposed to its influence. In those animals which have a double heart, and double circulation, lungs are used; in those that have a single heart, and a single circulating system,—under which head fall fishes and the *arachnida,* or spider class,—gills are the organs employed; while, in the incubated egg, oxygen exerts its influence on the blood through the medium of a membrane attached to the shell. Thus we see in those animals to whom respiration, or a process similar to it, is most necessary, that a diversity of means are used to effect the same purpose. But in these, and in all other animals in whom a circulation exists, a liver is found, composed of an aggregation of minute glands, through which a large portion of the blood, when loaded with impurities, is obliged to circulate.

I have said that in all animals that have a circulation a liver is found, and in no others does it exist; although they may have a stomach for the digestion of food; and in some of them, as will be shewn hereafter, even a fluid similar to bile is secreted.

Those animals which belong to the second and third classes, into which I have for convenience divided them; namely, those that can support life for a considerable time, and those that can live altogether, without the exposure of their blood to the influence of oxygen, shew most decidedly the important connexion that exists between the liver and the circulating system.

In the former of these—amphibious animals, and those birds that are good divers,—we find that, in proportion as they are able to suspend the process of respiration, the liver increases in size. Amongst birds, the Divers, or those arranged under the genus *Colymbus,* have the smallest lungs and the largest livers. The cormorant, also, (*Pelecanus Carbo,*) is an excellent diver, being able to suspend its respiration for a considerable time. Its liver is very large, while its lungs are comparatively small. In this bird, (as also in the other divers,) not only does the blood from the mesenteric, splenic, pancreatic, and gastric veins pass to the liver, but, from the very large vessels by which the mesenteric vein communicates with the veins emptying themselves into the cava and iliac veins, the blood passes with equal facility either through the vena portæ or the vena cava. So that when the bird is deprived of air, by diving, an additional quantity of blood is passed through the liver, in consequence of the pulmonary circulation being obstructed. In so small a bird as the *Fulica atra,* or common coot, I have, without difficulty, injected the system of the vena portæ from the iliac vein.*

The structure of amphibious animals shows a still more complete arrangement for the transmission of blood to the liver, when the respiration is obstructed. In them the liver is always very large. In some of them, as the frog, the circulation is carried on by a single heart; so that, when the lungs do not perform their functions, the obstruction of the circulation through the branches distributed to them does not interfere with the general circulation. In others, as the otter, although a double heart and circulation is found, a communication exists between the two sides of the heart, through the foramen ovale, so that when the pulmonary circulation ceases, the blood passing immediately from the right to the left side of the heart, a single circulation, like that in the frog, is immediately established.

In those animals that have the circulation carried on entirely without the blood being exposed to oxygen, the liver is proportionably larger than in any other. Thus, in the fœtus in utero, the liver is one of the first organs developed, and is by far the largest organ in the body. It is thus large, too, at a time when it should be the smallest, if it be true that the sole function of the liver is the secretion of bile to assist in digestion.

If the early formation of an organ be any proof of its great importance in early life; and if the service to which it is first devoted be any guide to the function it is ultimately to perform, the liver must be of the greatest importance to early life, and its function must be subservient to the circulation. The first of these assertions is proved by examining the incubated egg: for we find that the liver is developed as early as the fourth day of incubation, although at that time the heart is not developed, and the lungs do not appear until a day or two afterwards. That its function is subservient to the circulation, is proved by the veins first formed all terminating in the vena portæ; the circulating fluid being thus obliged to pass through the liver. As the gallbladder does not appear until a day or two later, the liver cannot be engaged at this time in the secretion of bile.

Having thus endeavoured to shew how closely the liver is connected with the circulation of the blood, I shall, in the

* It may be mentioned also as a curious fact that birds of the Struthious class, (as the ostrich and cassowary,) from the rapidity with which they run, and are therefore liable to have their respiration suspended, have also large livers and small lungs.

next place, endeavour to prove that the service it performs in the digestion of the food is but of secondary importance.

M. Cuvier has proved that the conglomerate glands which constitute the liver exist in all animals that have a circulation, and do not exist in any that have not a circulation, although in them digestion takes place. Amongst insects this fact is strongly marked. They are divided into two great classes; the *Arachnida,* comprising the spiders and scorpions, and the true *Insecta,* which comprise all other insects. In the former a circulating system is found, and also a liver: in the latter there is no circulating system, and no liver. What is particularly remarkable, and perhaps shews more decidedly than any other fact that the liver is subservient to the circulation, is this, that, although in the latter, there being no circulation, no liver exists, yet bile is secreted for the purposes of digestion from small, slender, filiform vessels, which empty their secretions into the alimentary canal. If the conglomerate glands constituting the liver in all animals having a circulation, be merely formed for the secretion of bile, why this deviation from the ordinary structure, which prevails in animals so closely resembling them in other respects, as the *Arachnida?* Moreover, we learn from the examination of some of the *Zoophytes,* that digestion may take place where a stomach only exists,—no liver or alimentary canal being present; the secretions from the stomach itself effecting all the necessary changes in the food.

Are we not, then, justified in concluding that, as we find digestion taking place in some animals without a liver, while in others bile is secreted from small vessels into the alimentary canal, this organ is but of secondary importance in the process of digestion.

On the other hand, are we not also justified in concluding that an organ, which is universally found where there is a circulating system, and there only, which is of comparatively small size where the respiration is perfectly performed by lungs, —which increases in size in those animals which have gills as an imperfect substitute for lungs,—which is found still larger in amphibious animals, whose circulation is occasionally suspended,—and which is the largest organ in the body in the fœtus, whose circulating fluid must be purified entirely without exposure to oxygen,—are we not, I say, justified in concluding that the primary function of such an organ must be similar to that of the lungs: that it must be subservient to the circulating system.

It may be objected that if, as I suppose, the liver does in amphibious animals perform the function of the lungs, these animals could live entirely without respiration. But how frequently do we see in the animal economy, when two organs are engaged in performing the same function, that one organ is able to perform almost the whole duty of the other, and yet cannot entirely dispense with its assistance. This is particularly the case with the skin and kidney. When one secretes copiously, the other enjoys comparative rest. From the length of time that some diving birds and amphibious animals remain under water, it is certain that death would result if the blood were not deprived of its impurities by some means; and certainly their anatomy shows that provision is made for the passage of an increased quantity of blood through the liver, that organ being proportionately increased in size to enable it to perform so important a function.

With regard to the function which I suppose it to perform in the fœtus, it may be said that the placenta purifies the blood. I grant the importance of the placenta; but we know that the principal change produced in the blood is the separation of carbon from it. This the placenta cannot effect, for it throws off nothing; while we know that the liver separates an amazing quantity of carbon during uterine life. This is proved by the quantity of carbon contained in the meconium; which secretion, as it cannot be of any use in the digestive process, can only be looked upon as a quantity of useless matter thrown off from the general system.

In what I have hitherto said it has been my object to point out, more particularly, the functions of the liver in amphibious animals and the fœtus. In them the other organs concerned in the purification of the blood being less perfect, the liver is found proportionably more developed. But, in animals that have a perfect respiration by lungs, even in man himself, we may observe many facts that point out the close connexion existing between this organ and the organs of respiration.

All who have observed the human subject must be aware that the liver is assisted by other organs in the performance of its function. For instance, the well known fact, that after severe burns, which have rendered a large portion of the skin unable to perform its function, persons will frequently die of suffocation: this shews that the lungs and skin both perform the same function, or so great an oppression of the lungs could not be produced by such a cause.

When a European is exposed to a tropical climate, where, from the state of the atmosphere, but little carbonic acid is formed during respiration, in what way do we find the circulating fluid purified? For a short time the skin, which is generally acknowledged to assist the lungs, acts profusely. Presently, however, the skin becomes dry and arid, and bile is secreted in such quantities as completely to disorder, instead of assisting, the digestive process.

It is true we cannot point out the manner in which the liver effects its changes in the circulating fluid, with the same precision that we can explain the function of the lungs; but, while there are so many circumstances connected with secretion that we are unable to account for, let us not presume to say that an effect cannot be produced because we cannot follow nature through every step of her process. Let us rather carefully observe the organs she finds necessary for effecting her different purposes, and acknowledge their use, though unable to account for their actions.

***Leamington Spa, Warwickshire;***
***August** 8th,* 1828.

P.S. Since the above paper was written, I have had much pleasure in reading a note by Dr. Elliotson, in the edition of Blumenbach's Physiology which he has recently published, in which he has mentioned many facts tending to support the same view of the function of the liver which I have endeavoured to advocate.

---

